# Effects of 8-week sprint interval training on cardiorespiratory fitness, blood lactate responses, stroke performance, and neuromuscular function in collegiate male tennis players

**DOI:** 10.3389/fphys.2026.1956423

**Published:** 2026-10-29

**Authors:** Yupeng Li, Bo Shi

**Affiliations:** 1 College of Physical Education and Health, Linyi University, Linyi, Shandong, China; 2 School of Recreation and Community Sport, Capital University of Physical Education and Sports, Beijing, China

**Keywords:** blood lactate, cardiorespiratory fitness, RSI, SIT, stroke accuracy, tennis

## Abstract

**Background:**

Tennis requires repeated high-intensity movement, rapid recovery, and stable stroke execution. This study compared court-based sprint interval training (SIT) with continuous endurance training (CT) for cardiorespiratory fitness, blood lactate responses, stroke performance, and neuromuscular ability in collegiate male tennis players.

**Methods:**

In this single-blind trial, forty-eight collegiate male tennis players were randomly allocated to SIT or CT (n = 24 per group) for 8 weeks, with three supervised conditioning sessions per week in addition to regular tennis training. Both groups attended regular tennis classes twice weekly for 45 min per session under the same course schedule. SIT consisted of court-based 110-m shuttle sprints (3 sets × 3 repetitions; 20 s between repetitions and 5 min between sets), whereas CT was treadmill running at 65% of baseline VO_2_max, progressing from 30 to 60 min per session. Cardiorespiratory fitness, blood lactate, standardized stroke performance, jumping and reactive-strength measures, 20-m sprint time, and 5-0–5 change-of-direction time were assessed before and after training. Interaction tests were adjusted using Holm’s method within six outcome families, with secondary outcomes interpreted exploratorily.

**Results:**

VO_2_max increased from 50.41 ± 1.62 to 53.24 ± 2.05 mL·kg^−1^·min^−1^ in SIT and from 49.83 ± 1.98 to 51.58 ± 2.44 mL·kg^−1^·min^−1^ in CT. VT2 increased from 2530.08 ± 136.72 to 2744.79 ± 205.49 mL·min^−1^ in SIT and from 2490.94 ± 189.47 to 2597.70 ± 222.72 mL·min^−1^ in CT. The group × time interactions remained significant after within-family Holm adjustment for VO_2_max (adjusted P = 0.023) and VT2 (adjusted P = 0.005). Exploratory interactions also remained significant within their respective families for stroke accuracy (adjusted P < 0.001), unforced-error rate (adjusted P = 0.009), RSI (adjusted P < 0.001), 20-m sprint time (adjusted P = 0.020), and 5-0–5 change-of-direction time (adjusted P = 0.007). BLa-15 and BLa-R15 estimates favored SIT, but neither interaction remained significant after adjustment (both adjusted P = .0.054).

**Conclusions:**

Court-based SIT produced greater improvements than CT in VO_2_max and VT2. Domain-adjusted exploratory findings also supported greater improvements in stroke performance, RSI, sprint performance, and change-of-direction performance, whereas recovery-phase blood lactate differences remained uncertain after adjustment.

## 1 Introduction

Coaches must fit conditioning alongside technical–tactical practice and recovery within a finite training schedule. The practical question is how to develop the capacity for repeated intense efforts without requiring lengthy additional conditioning sessions or compromising the quality of tennis practice. Tennis combines acceleration, braking, directional changes, and stroke production with recovery between points; conditioning therefore needs to support both sustained aerobic work and repeated explosive actions (Bergeron et al., 1991; Kovacs, 2007; Reid et al., 2008). Continuous endurance training remains a useful aerobic stimulus, but its uninterrupted movement pattern does not directly reproduce these demands. The rationale for examining alternatives is their potential to combine aerobic conditioning with a more relevant movement stimulus and a shorter conditioning component, rather than min an assumption that continuous running has become ineffective or has been while universally abandoned (Helgerud et al., 2007; Zhao et al., 2024; Yang et al., 2025).

High-intensity interval training (HIIT) can improve aerobic fitness in tennis players, and on-court drills can provide a substantial physiological stimulus (Reid et al., 2008; Fernandez-Fernandez et al., 2017; Kilit and Arslan, 2019). However, HIIT describes varied protocols: a longer running interval may train aerobic capacity without reproducing maximal accelerations, repeated braking, or stroke execution. Its transfer to tennis therefore depends on exercise mode, intensity, and recovery structure. Here, sprint interval training (SIT) denotes repeated maximal-effort sprints, whereas conventional HIIT refers to submaximal high-intensity intervals; SIT may also be classified within the broader HIIT family. Acute physiological responses differ between these formats (Wood et al., 2016). Court-based SIT offers a plausible way to combine an aerobic stimulus with repeated directional movements, but these potential advantages require direct testing and do not establish superiority over all HIIT protocols. Repeated-sprint training has also been examined in competitive tennis players (Fernandez-Fernandez et al., 2012), including a hypoxic-training case study in a rookie professional player (Brechbuhl et al., 2018).

The proposed framework links the training stimulus to two complementary adaptation pathways. First, repeated intense exercise while can increase muscle oxidative capacity, providing a physiological basis for improved aerobic performance and recovery (Gibala et al., 2006; Burgomaster et al., 2008). Maximal oxygen uptake (VO_2_max) reflects integrated oxygen delivery and utilization, while the second ventilatory threshold (VT2) characterizes the exercise intensity associated with respiratory compensation. Greater oxidative capacity could support oxygen-dependent phosphocreatine resynthesis between efforts and lactate utilization. Human studies associate trained status with greater lactate disposal and relate muscle monocarboxylate transporter content to post-exercise blood lactate removal (Thomas et al., 2005; Messonnier et al., 2013). An increase in VO_2_max is therefore a complementary indicator of adaptation, not itself a direct cause or proof of faster lactate clearance.

Blood lactate concentration reflects the balance between lactate appearance and disposal, rather than clearance alone (Messonnier et al., 2013). Concentration at 15 min of recovery (BLa-15) and the relative decline from the observed peak (BLa-R15) can describe the post-exercise response, but neither directly measures lactate flux. A possible functional link is that better recovery between intense efforts helps preserve court positioning and movement coordination, which may support accurate ball contact. Fatigue can impair skilled tennis performance (Davey et al., 2002), but lactate should not be treated as a direct cause of technical errors. Thus, recovery-related preservation of late-set accuracy is a testable hypothesis, not an program established consequence of a lower blood lactate value. In this study, lactate was sampled after treadmill exercise and stroke performance was assessed separately; these measurements cannot establish that lactate changes mediate technical improvements.

Second, repeated acceleration and min braking may provide a neuromuscular stimulus that supports rapid force production and repositioning. Jump and reactive-strength tests characterize this pathway alongside the aerobic and metabolic measures. Countermovement jump (CMJ), squat jump (SJ), their height ratio (eccentric utilization ratio, EUR), and drop-jump reactive strength index (RSI; jump height divided by contact time) sample different aspects of lower-limb performance. Standardized procedures are important for their reliability (Flanagan et al., 2008; Markwick et al., 2015; Carroll et al., 2019; Comyns et al., 2019; Heishman et al., 2020). A combined repeated-sprint and explosive-strength intervention improved movement and jump performance in junior tennis players (Fernandez-Fernandez et al., 2015), but the contribution of sprint training alone cannot be isolated from that combined program. RSI improvement is therefore considered a possible parallel adaptation, not a prerequisite for accurate stroke production or evidence of its cause. Accuracy and unforced-error rate assess technical execution directly, while jump, sprint, and min change-of-direction tests characterize physical capacities that may support it.

Previous tennis studies provide relevant controlled evidence, but answer different questions. Yang et al. randomized 16 players to six weeks of SIT or continuous endurance training and assessed aerobic fitness, recovery-phase lactate, and endurance tests (Yang et al., 2025). Zhao et al. randomized 24 players to comparable training categories and examined anaerobic and repeated-sprint performance, including lactate responses (Zhao et al., 2024). Fan et al. randomized 30 players to eight weeks of SIT or HIIT and included physiological and stroke-performance measures (Fan et al., 2025). These studies therefore already used active comparators and covered more than one domain. Their outcome sets did not jointly include the recovery-phase, technical, and reactive-strength assessments used here, but adding measures alone is not a sufficient scientific rationale.

Methodological qualifications are also relevant. Yang et al. and Zhao et al. reported *a priori* power calculations based on a large effect (f = 0.40) and three measurements, although their described intervention comparisons used PRE and POST assessments (Zhao et al., 2024; Yang et al., 2025). Those assumptions limit inference about sensitivity to smaller effects in the reported design. Fan et al. reported *post hoc* power based on an observed effect, which does not establish prospective sensitivity across outcomes (Fan et al., 2025). None of these three reports explicitly described assessor blinding in the published methods; this is a reporting limitation, not proof that blinding was absent. Their findings remain informative, but small group sizes, outcome-specific power assumptions, and incomplete blinding information warrant caution when interpreting broad adaptation claims.

The unresolved question is whether the aerobic benefit of a practical court-based sprint program is accompanied by favorable recovery-phase, technical, and program neuromuscular responses in the same players, or whether adaptation is confined to selected capacities. Joint assessment is useful because improved aerobic fitness alone cannot demonstrate better stroke execution or reactive strength, and technical gains need not depend on measurable changes in lactate recovery. This framework predicts potentially complementary, rather than obligatorily coupled, responses. It supports an active-comparator trial with a defined cardiorespiratory question and exploratory assessment of the other domains, while distinguishing a training-effect comparison from a causal test of program the proposed physiological pathways.

Accordingly, this single-blind, randomized, parallel-group trial compared eight weeks of court-based SIT program with continuous treadmill endurance training (CT), each performed three times weekly alongside the same scheduled regular tennis training, in 48 collegiate male tennis players. VO_2_max, maximal ventilation (VEmax), and VT2 were the primary cardiorespiratory outcomes. Exploratory secondary outcomes organize covered additional ventilatory indices, blood lactate responses, stroke performance, neuromuscular performance, and field-based physical performance. This structure retained the primary aerobic-conditioning question while examining the broader response predicted by the framework. The active comparator addresses the choice between two conditioning programs; without a tennis-training-only group or a HIIT arm, the study cannot isolate the effect of adding conditioning or compare SIT directly with conventional HIIT.

We hypothesized that SIT would produce greater improvements in primary cardiorespiratory outcomes than CT. Based on the proposed oxidative-recovery and neuromuscular pathways, we also explored whether SIT would produce more favorable recovery-phase lactate responses, stroke accuracy and while unforced-error rates, and selected lower-limb performance outcomes. These secondary hypotheses concern changes in separate outcome domains; they do not predict that every measure must improve or that changes in VO_2_max, lactate, RSI, and stroke performance must follow a proven causal sequence.

## 2 Methods

### 2.1 Study design and ethical approval

This study used an 8-week, single-blind, two-arm, parallel-group randomized controlled design to compare the effects of court-based sprint interval training (SIT) and continuous endurance training control (CT) on cardiorespiratory fitness, blood lactate responses, standardized tennis stroke performance, neuromuscular capacity, and selected physical-performance outcomes in male collegiate tennis players.

Forty-eight participants were randomly allocated in a 1:1 ratio to the SIT group (n = 24) or CT group (n = 24). Assessments were performed before the intervention (PRE) and after completion of the 8-week intervention (POST). The study was approved by the Ethics Committee of Capital University of Physical Education and Sports, Beijing, China (Approval No. 2025A385). All procedures conformed to the Declaration of Helsinki, and written informed consent was obtained from every participant before participation.

### 2.2 Participants

Forty-eight male collegiate tennis players were recruited from Capital University of Physical Education and Sports. In accordance with the Participant Classification Framework proposed by McKay et al. (2022), all participants were classified as Tier 3 athletes (highly trained/national-level). This classification was based on their sustained sport-specific training history, established competitive tennis background, and participation in high-level Chinese collegiate tennis competition.

Participants were eligible if they were 18–23 years of age, had accumulated at least 5 years of systematic tennis training, and had participated in Chinese Collegiate Tennis Competition Group A events during the preceding 12 months. Participants were excluded if they had experienced a lower-limb musculoskeletal injury, interruption of systematic training, or substantial illness during the preceding 3 months; had known cardiovascular, metabolic, or respiratory disease; used medication that could influence exercise capacity or blood lactate responses; or were unable to safely complete maximal running, sprinting, jumping, and tennis stroke-performance tests.

*A priori* sample-size estimation was performed using G*Power version 3.1 (Heinrich-Heine-Universität Düsseldorf, Germany) for an F test of the group × time interaction in a two-way mixed-design analysis of variance. Input parameters were: effect size f = 0.25 (medium interaction effect), α = 0.05, desired power = 0.80, number of groups = 2, number of repeated measurements = 2, correlation among repeated measures = 0.5, and nonsphericity correction ϵ = 1.0. Under these assumptions, a total of 36 participants (18 per group) was required. The final sample comprised 48 participants, with 24 participants allocated to each group, and therefore exceeded the *a priori* minimum sample-size. requirement. This calculation concerned an assumed interaction effect and did not establish adequate power for every secondary outcome or for a mediation analysis. Baseline characteristics are reported in **Table 1**.

**Table 1:** Baseline characteristics of participants.

Variable	SIT (n = 24)	CT (n = 24)	P value
Age, years	20.6 ± 1.3	20.8 ± 1.2	0.58
Height, cm	178.5 ± 5.4	177.9 ± 5.7	0.71
Body mass, kg	72.8 ± 6.9	72.1 ± 6.5	0.72
Body mass index, kg·m^−2^	22.9 ± 1.7	22.8 ± 1.6	0.84
Systematic tennis training, years	6.3 ± 1.4	6.1 ± 1.5	0.64

Values are mean ± SD.

### 2.3 Randomization, familiarization, and experimental overview

After baseline assessment, participants were assigned identification numbers and randomly allocated to SIT or CT using a computer-generated random sequence in SPSS Statistics. The randomization sequence was prepared by a researcher who was not involved in outcome assessment. The study was single-blind with respect to outcome assessment: participants and training supervisors were aware of allocation, whereas laboratory assessors and video scorers were blinded to group assignment. Statistical analysts were also blinded to group assignment. These role-specific procedures describe the scope of blinding; participants were not blinded to the visibly different training programs.

All participants completed two familiarization sessions before PRE testing. Familiarization included the court-based shuttle-running route, treadmill exercise while wearing the metabolic mask, jump-testing procedures, linear sprint and change-of-direction testing, and the standardized stroke-accuracy protocol.

The PRE and POST batteries were completed over three visits separated by at least 48 h. The POST battery began 48 h after the final intervention session and followed the identical order and procedures used at PRE. Visit 1 included anthropometry, CMJ, SJ, drop jump, and standardized tennis stroke-performance testing. Visit 2 included the 20-m sprint, 5-0–5 change-of-direction test, and standing long jump. Visit 3 included the graded cardiopulmonary exercise test and serial blood lactate sampling.

Participants were instructed to avoid strenuous exercise for 48 h, alcohol for 24 h, and caffeine for 12 h before each testing visit. They were asked to maintain their habitual diet and sleep patterns throughout the study and consume a similar meal 3–4 h before PRE and POST testing. Both groups continued their scheduled regular tennis classes during the intervention, as described in Section 2.4.

### 2.4 Training intervention

The intervention lasted 8 weeks. Both groups continued their regular university tennis training classes twice weekly for 45 min per session, corresponding to 90 min of scheduled tennis training per week, under the same course schedule. In addition, participants completed three supervised conditioning sessions per week according to their group allocation: court-based SIT on Monday, Wednesday, and Friday, or continuous treadmill endurance training (CT) on Tuesday, Thursday, and Saturday. Thus, the study compared regular tennis training plus SIT with regular tennis training plus CT; no regular-tennis-training-only group was included.

Both groups completed the same standardized warm-up before training. The warm-up lasted approximately 20 min and consisted of 8 min of low-intensity jogging, dynamic quadriceps stretching, two 10-m bouts of butt-kick running, two 10-m bouts of high-knee running, light calf stretching, lateral lunge walking, and dynamic whole-body mobility exercises. After training, participants completed 5–6 min of low-intensity walking followed by approximately 15 min of static stretching and recovery activities. The complete 8-week progression and standardized within-session structure are summarized in **Tables 2** and **3**. The intervention was developed with reference to independent English-language trials of sprint interval and continuous endurance training (Macpherson et al., 2011; Litleskare et al., 2020; Jin et al., 2025). All intervention sessions were supervised by the same four-member team throughout the 8-week study, consisting of two coaches and two graduate research assistants. The same personnel were involved in both the SIT and CT groups from the beginning to the end of the intervention. The coaches were primarily responsible for implementing the prescribed training procedures, providing standardized instructions, and monitoring exercise execution, whereas the graduate research assistants assisted with session organization, physiological monitoring, and documentation of protocol completion and any deviations. All supervisors followed the same standardized intervention procedures and monitoring criteria throughout the study to ensure consistency between groups and minimize potential investigator-related bias.

**Table 2:** Eight-week intervention program for the SIT and CT groups.

Phase	SIT group	CT group
Weeks 1–2	3 sessions·wk^−1^ (Monday, Wednesday, and Friday); 3 sets × 3 maximal shuttle repetitions; 110 m per repetition (990 m·session^−1^). Shuttle route: 0–20–5–25–5–10–5–15–0 m. Recovery: 20 s between repetitions and 5 min between sets.	3 sessions·wk^−1^; continuous treadmill running at the speed corresponding to 65% baseline VO_2_max; 30 min·session^−1^.
Weeks 3–4		3 sessions·wk^−1^; continuous treadmill running at the speed corresponding to 65% baseline VO_2_max; 45 min·session^−1^.
Weeks 5–8		3 sessions·wk^−1^; continuous treadmill running at the speed corresponding to 65% baseline VO_2_max; 60 min·session^−1^.

**Table 3:** Standardized within-session structure, dose, and fidelity procedures.

Session phase	SIT group	CT group
0–8 min: General warm-up	Low-intensity jogging.	Low-intensity jogging.
8–20 min: Dynamic preparation	Dynamic quadriceps stretching; 2 × 10-m butt-kick runs; 2 × 10-m high-knee runs; light calf stretching; lateral lunge walking; and whole-body mobility.	Identical standardized dynamic preparation.
20–25 min: Transition	Hydration as required; Polar sensor check; route review and positioning at the 0-m cone.	Hydration as required; Polar sensor check; individual treadmill speed confirmation and set-up.
Main intervention	3 sets × 3 maximal 110-m shuttle repetitions; 20-s recovery between repetitions and 5-min recovery between sets (9 repetitions; 990 m per session; ≈16–18 min including scheduled recovery).	Continuous treadmill running at the speed corresponding to 65% baseline VO_2_max: 30 min in weeks 1–2, 45 min in weeks 3–4, and 60 min in weeks 5–8.
Immediately after main block	5–6 min low-intensity walking.	5–6 min low-intensity walking.
Recovery activities	≈15 min standardized static stretching and recovery activities.	≈15 min standardized static stretching and recovery activities.
Monitoring and fidelity	Polar Team Pro: mean/peak HR, time in HR zones, and effective intervention time. Attendance, route completion, protocol deviations, illness, and injuries recorded.	Polar Team Pro: mean/peak HR, time in HR zones, and effective intervention time. Attendance, protocol deviations, illness, and injuries recorded.

**Table 3** separates common preparation and recovery from the group-specific training stimulus. The exercise content and dose were derived from the tennis-specific training protocol and contextualized by independent sprint-interval and continuous-training trials (Macpherson et al., 2011; Litleskare et al., 2020; Jin et al., 2025).

#### 2.4.1 Sprint interval training group

The SIT protocol was adapted from the court-based shuttle-running protocol used in the preceding master’s thesis. Cones were placed at 0, 5, 10, 15, 20, and 25 m. One shuttle repetition followed the sequence 0 m → 20 m → 5 m → 25 m → 5 m → 10 m → 5 m → 15 m → 0 m, corresponding to a total running distance of 110 m. Each repetition was completed at maximal effort, with participants instructed to accelerate, decelerate, and change direction as rapidly as possible while completing the prescribed route.

Each SIT session comprised three sets of three maximal shuttle repetitions. Recovery duration was 20 s between repetitions and 5 min between sets. Thus, participants completed nine 110-m shuttle repetitions in each session. Standardized verbal encouragement was provided throughout all sessions. If a participant failed to complete the prescribed route because of a technical error or interruption, the repetition was repeated after the scheduled recovery period.

#### 2.4.2 Continuous endurance training control group

The CT group performed continuous treadmill running at a speed corresponding to 65% of each participant’s baseline VO_2_max. The individual running speed corresponding to 65% VO_2_max was determined from the PRE graded exercise test. Training duration progressed from 30 min per session during weeks 1–2, to 45 min per session during weeks 3–4, and to 60 min per session during weeks 5–8. Treadmill speed was adjusted when necessary to maintain the prescribed physiological intensity.

### 2.5 Training-load monitoring and adherence

Internal training load was monitored during every intervention session using the Polar Team Pro system with Polar Pro chest-worn sensors (Polar Electro Oy, Kempele, Finland). Individual maximal heart rate was obtained from the PRE graded exercise test. Heart-rate monitoring was used to document training intensity and supervise participant safety; heart-rate variables were not treated as intervention efficacy outcomes.

For each session, mean heart rate, peak heart rate, effective training time, and time spent in five heart-rate zones (50–60%, 60–70%, 70–80%, 80–90%, and 90–100% of maximal heart rate) were recorded. Effective training time was defined as the intervention period excluding warm-up and recovery stretching. Training impulse was calculated using the Edwards method: TRIMP = Σ(time in each heart-rate zone × zone weighting), with weighting factors of 1, 2, 3, 4, and 5 for Zones 1–5, respectively. Training attendance, missed sessions, illness, injury, and modified sessions were documented. Completion of at least 85% of prescribed sessions was considered acceptable adherence. For SIT, the completion time of each 110-m shuttle-sprint repetition was recorded. Mean repetition time and mean speed were calculated across completed repetitions, and sprint decrement was calculated as [Σ repetition times/(best repetition time × number of completed repetitions) − 1] × 100. Session-level monitoring variables were first averaged within each participant across attended sessions and were then summarized descriptively across participants.

### 2.6 Graded cardiopulmonary exercise test

Cardiorespiratory fitness was evaluated using a motorized treadmill (pulsar 3p, h/p/cosmos sports & medical GmbH, Nussdorf-Traunstein, Germany) coupled with a breath-by-breath metabolic system (Quark CPET, COSMED, Rome, Italy). The metabolic system was calibrated before each test according to the manufacturer’s instructions using room air, reference gas, and a 3-L syringe.

After a 5–10 min warm-up, the treadmill test began at 6 km·h^−1^ and speed increased by 1 km·h^−1^ every minute until 16 km·h^−1^ was reached. Thereafter, treadmill incline increased by 1.5% every minute until volitional exhaustion. VO_2_max was accepted when at least three of the following criteria were satisfied: heart rate ≥180 beats·min^−1^; a plateau in oxygen uptake despite increasing workload; respiratory exchange ratio ≥1.15; and volitional exhaustion. The highest 30-s averaged values were used to determine VO_2_max and maximal minute ventilation (VEmax).

The second ventilatory threshold (VT2) was identified using combined visual inspection of the V-slope method, a nonlinear increase in VE/VCO_2_, and the respiratory compensation response. The VE/VCO_2_ slope was calculated by linear regression of minute ventilation against carbon dioxide output during the incremental phase before respiratory compensation. The VT2/VO_2_max ratio was calculated as VT2 (mL·kg^−1^·min^−1^)/VO_2_max (mL·kg^−1^·min^−1^) × 100.

### 2.7 Blood lactate assessment

Capillary blood lactate concentration was assessed using an enzymatic lactate analyzer (Biosen C-Line, EKF Diagnostics, Cardiff, United Kingdom). Participants remained seated for 10 min before collection of the resting fingertip capillary blood sample. Additional samples were obtained immediately after the graded exercise test and at 1, 3, 5, 7, 10, and 15 min of passive recovery. After fingertip disinfection and drying, the first drop of blood was discarded and approximately 20 μL of capillary blood was analyzed.

Derived outcomes included resting blood lactate concentration (BLa-rest), peak post-exercise blood lactate concentration (BLa-peak; the highest value recorded from immediately after exercise to 10 min of recovery), blood lactate concentration at 15 min of recovery (BLa-15), and the 15-min relative reduction in blood lactate concentration (BLa-R15). BLa-R15 was calculated as [(BLa-peak − BLa-15)/BLa-peak] × 100. These concentration-based outcomes characterize recovery after the graded test and do not directly quantify lactate production, disposal, or metabolic clearance rate.

### 2.8 Standardized tennis stroke-performance test

Stroke performance was assessed using a standardized ball-machine-based groundstroke test on an indoor hard court. A programmable tennis ball machine (Spinshot Player, Spinshot Sports, Sydney, Australia) delivered balls with fixed speed, spin, trajectory, and frequency. After a 10-min tennis-specific warm-up and 10 familiarization feeds, participants completed 40 valid groundstrokes: 20 forehands and 20 backhands. Strokes were delivered in four alternating blocks of 10 balls.

Balls were delivered at 45 km·h^−1^ with moderate topspin and a 6-s inter-ball interval. Participants started each stroke from a marked central recovery position behind the baseline and were instructed to return the ball toward a predefined target zone on the opposite court. The target zone measured 1.5 × 1.5 m and was positioned approximately 1.0 m inside the baseline and singles sideline. A 2-min passive rest interval was provided after 20 strokes.

All trials were recorded using two high-definition cameras (GoPro HERO13 Black, GoPro Inc., San Mateo, CA, USA) positioned behind the baseline and laterally to the court. Two blinded assessors independently scored all recordings, and discrepancies were resolved by joint video review. Overall stroke accuracy was calculated as target hits/valid ball feeds × 100. Unforced-error rate was calculated as balls hit into the net, outside the singles court, or failing to cross the net/valid ball feeds × 100. Balls landing within the singles court but outside the target zone were classified as valid but inaccurate returns. Forehand and backhand accuracy were retained as descriptive component outcomes.

### 2.9 Neuromuscular-performance assessment

Neuromuscular performance was assessed using a three-component force platform (Kistler 9286AA, Kistler Instruments AG, Winterthur, Switzerland) sampled at 1000 Hz. The force platform was calibrated and zeroed according to the manufacturer’s instructions before each testing session. Ground-reaction-force data were collected and processed using Kistler Bioware software (Kistler Instruments AG, Winterthur, Switzerland).

Following a standardized warm-up, participants completed countermovement jump (CMJ), squat jump (SJ), and drop-jump assessments. Three valid trials were performed for each jump modality, with 60 s of passive recovery between trials and 3 min of recovery between jump modalities. Participants kept their hands on their hips during CMJ and SJ testing to minimize the influence of arm swing.

For CMJ, participants began from an upright standing position, performed a rapid countermovement to a self-selected depth, and immediately completed a maximal vertical jump. For SJ, participants adopted a static squat position with approximately 90° knee flexion, maintained this position for 3 s, and then performed a maximal vertical jump without a preparatory countermovement.

For the drop jump, participants stepped from a 30-cm box onto the force platform and immediately performed a maximal vertical jump with the shortest possible ground-contact time. Participants were instructed to step, rather than jump, from the box. Jump height was calculated from vertical take-off velocity using the impulse-momentum method. Reactive strength index (RSI) was calculated as jump height divided by ground-contact time. The neuromuscular outcomes were CMJ height (cm), SJ height (cm), RSI (m·s^−1^), and eccentric utilization ratio (EUR), calculated as CMJ height/SJ height. The best valid trial was retained for analysis; trials with arm swing, incorrect starting position, loss of balance, or an obvious technical error were repeated.

### 2.10 Additional physical-performance tests

#### 2.10.1 Twenty-meter sprint

Linear sprint performance was assessed using photocell timing gates (Witty, Microgate, Bolzano, Italy). Participants started 0.3 m behind the first timing gate to avoid premature triggering. They completed three maximal 20-m sprints from a standing start, with 3 min of passive recovery between trials. The fastest time was used for analysis.

#### 2.10.2 5-0–5 change-of-direction test

The 5-0–5 test was performed using the same timing-gate system. Participants sprinted forward for 15 m, crossed the 10-m timing gate, continued to the 15-m turning line, performed a 180° turn, and sprinted back through the 10-m gate. The timed interval therefore represented the 5-m approach, 180° turn, and 5-m return phase. Participants completed two trials turning from each limb, with 3 min of recovery between trials. The mean of the fastest valid trial from each turning direction was used for analysis; lower completion time indicated better change-of-direction performance.

#### 2.10.3 Standing long jump

Standing long-jump performance was assessed on a non-slip surface using a tape measure. Participants stood behind a marked line and performed a maximal bilateral horizontal jump using a self-selected arm swing. Three trials were completed with 2 min of recovery between trials. The longest valid distance was recorded to the nearest 1 cm.

### 2.11 Statistical analysis

All statistical analyses were performed using SPSS Statistics version 29.0 (IBM Corp., Armonk, NY, USA). Data are presented as mean ± standard deviation (SD). Normality was assessed using the Shapiro–Wilk test and visual inspection of Q–Q plots. Homogeneity of variance between groups was examined using Levene’s test. Because the within-subject factor included only two time points, sphericity correction was not required.

Baseline between-group comparability was examined using independent-samples t-tests. All outcomes, including cardiopulmonary variables, blood lactate measures, stroke-performance variables, neuromuscular measures, and additional physical-performance outcomes, were analyzed using two-way mixed-design analysis of variance, with group (SIT vs. CT) as the between-subject factor and time (PRE vs. POST) as the within-subject factor.

The group × time interaction was the principal test of differential change between groups. VO_2_max, VEmax, and VT2 were the primary cardiorespiratory outcomes; the remaining outcomes were exploratory secondary outcomes. To address multiplicity, the 18 interaction tests were organized into six conceptually defined outcome families: primary cardiorespiratory outcomes, additional ventilatory indices, blood lactate responses, stroke performance, neuromuscular performance, and field-based physical performance. Holm adjustment was applied separately within each family. Family membership and both unadjusted and adjusted interaction P values are reported in **Supplementary Table S2**. The adjusted P values control multiplicity within each defined family. Secondary outcomes were interpreted exploratorily irrespective of their adjusted statistical significance. When an unadjusted group × time interaction or main effect was identified at P < 0.05, follow-up comparisons were performed using paired-samples t-tests for within-group PRE-to-POST comparisons and independent-samples t-tests for between-group comparisons at POST, with Bonferroni adjustment applied to the follow-up comparisons. For outcomes with an unadjusted interaction P value < 0.05 but a within-family Holm-adjusted P value ≥ 0.05, the follow-up comparisons were interpreted as exploratory and were not used to establish statistically significant differential change between groups.

Effect sizes for analysis-of-variance effects were expressed as partial eta squared (partial η²), and standardized between-group differences at POST were expressed as Cohen’s d. Between-group differences in PRE-to-POST change were additionally reported in the original measurement units with unadjusted 95% confidence intervals. Differences in change were calculated as the PRE-to-POST change in SIT minus that in CT. Effect estimates were interpreted according to their direction, magnitude, and precision. All tests were two-tailed. Group × time interactions were evaluated using within-family Holm-adjusted P < 0.05.

## 3 Results

Unadjusted interaction P values are retained in the outcome-specific results, with the corresponding within-family Holm-adjusted values reported in **Supplementary Table S2**. After adjustment, interactions remained significant for VO_2_max, VT2, stroke accuracy, unforced-error rate, RSI, 20-m sprint time, and 5-0–5 change-of-direction time. BLa-15 and BLa-R15 did not remain significant (both adjusted P = 0.054), nor did the VE/VCO_2_ slope (adjusted P = 0.099). Secondary findings remain exploratory.

### 3.1 Cardiopulmonary fitness and ventilatory efficiency

The group × time interactions for V̇O_2_max and VT2 remained significant after Holm adjustment within the primary cardiorespiratory family. V̇O_2_max increased from 50.41 ± 1.62 to 53.24 ± 2.05 mL·kg^−1^·min^−1^ in SIT and from 49.83 ± 1.98 to 51.58 ± 2.44 mL·kg^−1^·min^−1^ in CT, with a greater improvement following SIT (F(1,46) = 6.90, unadjusted P = 0.012, adjusted P = 0.023, partial η² = 0.130). At POST, V̇O_2_max was higher in SIT than in CT (P = 0.014, Cohen’s d = 0.74; **Figure 1A**).

**Figure 1:**
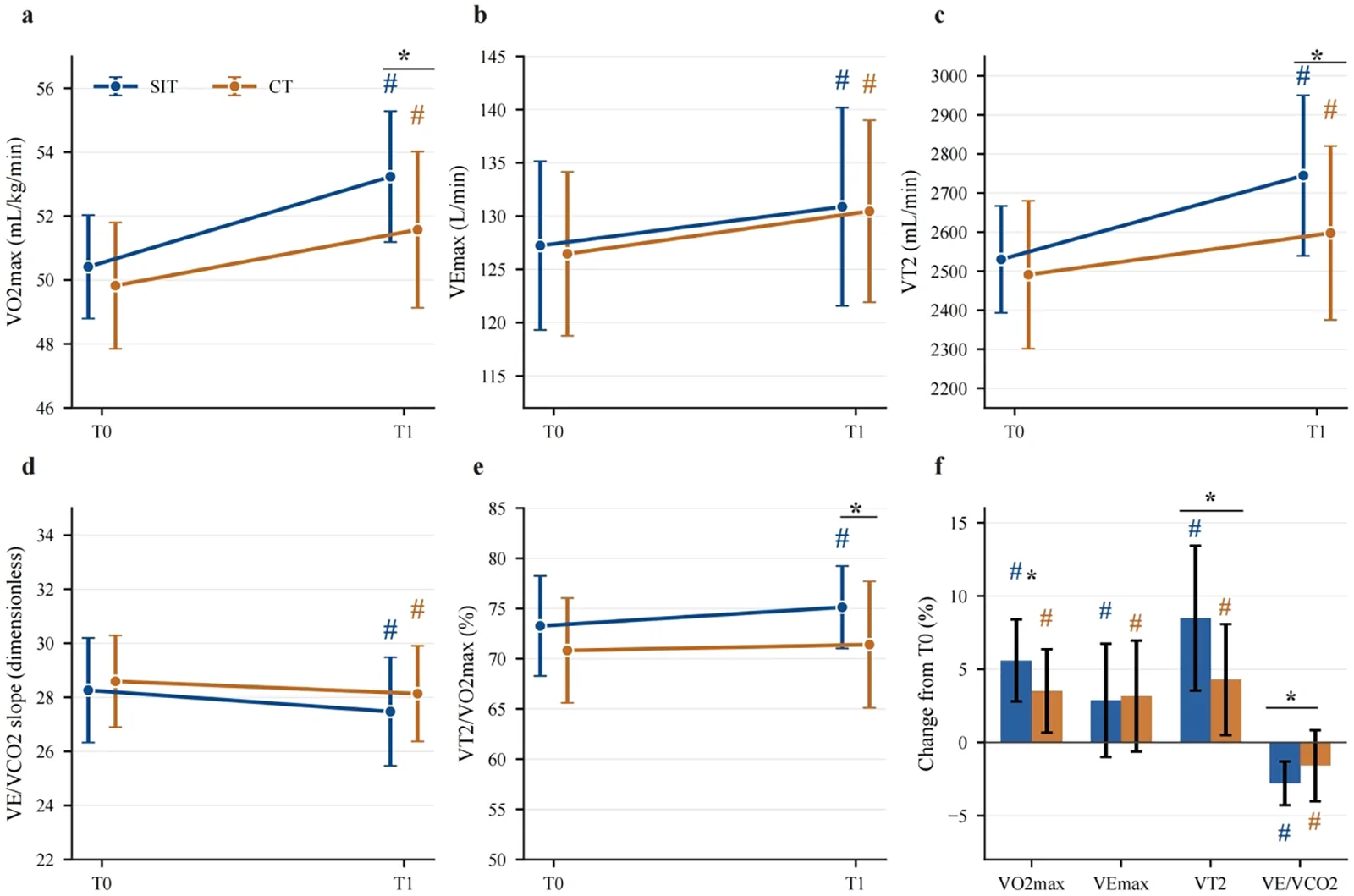
Cardiopulmonary fitness and ventilatory efficiency before and after the 8-week intervention. Data are presented as mean ± SD (n = 24 per group). The SIT group completed court-based 110-m shuttle sprints (3 sets × 3 repetitions; 20 s between repetitions and 5 min between sets) three times weekly. The CT group completed three weekly treadmill continuous-running sessions at the individual speed corresponding to 65% of baseline V̇O_2_max, progressing from 30 to 60 min across the intervention. Panels show **(a)** maximal oxygen uptake (VO_2_max), **(b)** maximal minute ventilation (VEmax), **(c)** the second ventilatory threshold (VT2), **(d)** the VE/VCO₂ slope, **(e)** the VT2/VO₂max ratio, and (f) percentage changes from baseline in VO₂max, VEmax, VT2, and the VE/VCO_2_ slope. #P < 0.05 versus PRE within the same group; *P < 0.05 for SIT versus CT at POST, where shown. Domain-specific Holm-adjusted group × time interaction results are presented in **Supplementary Table S2**. These interaction tests are distinct from the within-group and POST between-group comparisons indicated by the symbols.

VT2 increased from 2530.08 ± 136.72 to 2744.79 ± 205.49 mL·min^−1^ in SIT, compared with an increase from 2490.94 ± 189.47 to 2597.70 ± 222.72 mL·min^−1^ in CT (F(1,46) = 11.38, unadjusted P = 0.002, adjusted P = 0.005, partial η² = 0.198). The POST VT2 value was also greater in SIT than in CT (P = 0.022, d = 0.69; **Figure 1C**).

Both groups increased VEmax over time (F(1,46) = 29.78, P < 0.001, partial η² = 0.393); however, the group × time interaction was not significant (F(1,46) = 0.06, unadjusted P = 0.804, adjusted P = 0.804, partial η² = 0.001; **Figure 1B**). The VE/V̇CO_2_ slope interaction was significant before adjustment but did not remain significant after Holm adjustment within the additional ventilatory family (F(1,46) = 4.07, unadjusted P = 0.049, adjusted P = 0.099, partial η² = 0.081). The POST between-group comparison was not significant (P = 0.230, d = −0.35; **Figure 1D**). The VT2/V̇O_2_max ratio also showed limited evidence of differential change (F(1,46) = 2.84, unadjusted P = 0.098, adjusted P = 0.099, partial η² = 0.058; **Figure 1E**). Percentage changes from baseline in V̇O₂max, VEmax, VT2, and the VE/V̇CO_2_ slope are shown in **Figure 1F**.

### 3.2 Blood lactate responses and recovery

Blood lactate results are presented as resting concentration (**Figure 2A**), peak post-exercise concentration (**Figure 2B**), concentration at 15 min of recovery (**Figure 2C**), the relative reduction from peak blood lactate to 15 min of recovery (**Figure 2D**), and the recovery time course (**Figure 2E**). BLa-15 decreased from 6.70 ± 0.65 to 5.44 ± 1.05 mmol·L^−1^ in SIT and from 6.59 ± 0.70 to 6.08 ± 1.21 mmol·L^−1^ in CT. The interaction was significant before adjustment but did not remain significant after Holm adjustment within the lactate family (F(1,46) = 6.21, unadjusted P = 0.016, adjusted P = 0.054, partial η² = 0.119; **Figure 2C**). BLa-R15 increased from 33.97 ± 4.06% to 45.78 ± 10.89% in SIT and from 34.20 ± 3.95% to 38.43 ± 10.82% in CT. This interaction also did not remain significant after adjustment (F(1,46) = 6.60, unadjusted P = 0.014, adjusted P = 0.054, partial η² = 0.125). The effect estimates for both recovery-phase measures favored SIT.

**Figure 2:**
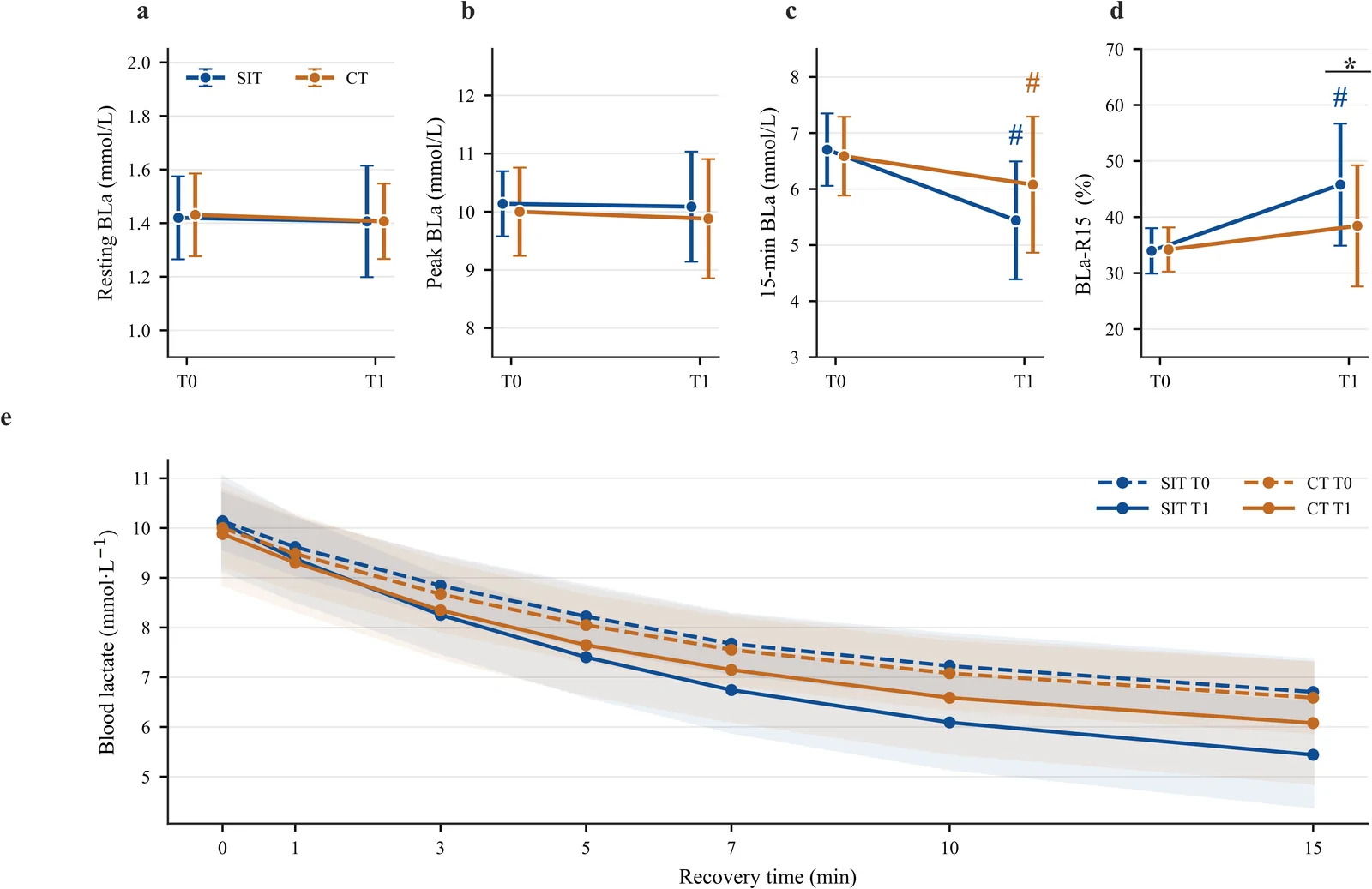
Blood lactate responses and post-exercise recovery before and after the 8-week intervention. Data are presented as mean ± SD (n = 24 per group). Panels show **(a)** resting blood lactate concentration, **(b)** peak post-exercise blood lactate concentration, **(c)** blood lactate concentration at 15 min of recovery (BLa-15), **(d)** the relative reduction from peak blood lactate to 15 min of recovery (BLa-R15), and **(e)** the blood lactate recovery time course at T0 and T1. The recovery trajectory is shown at rest, immediately after exercise, and 1, 3, 5, 7, 10, and 15 min of passive recovery. BLa-R15 was calculated as [(BLa-peak −BLa-15)/BLa-peak] × 100. #P < 0.05 versus PRE within the same group; *P < 0.05 for SIT versus CT at POST, where shown. Domain-specific Holm-adjusted group × time interaction results are presented in **Supplementary Table S2**. These interaction tests are distinct from the within-group and POST between-group comparisons indicated by the symbols.

### 3.3 Standardized tennis stroke performance

Overall stroke accuracy showed the largest interaction effect-size estimate among the study outcomes, and the interaction remained significant after Holm adjustment within the stroke-performance family (F(1,46) = 23.22, unadjusted P < 0.001, adjusted P < 0.001, partial η² = 0.335). Accuracy increased from 64.01 ± 5.21% to 68.23 ± 5.63% in SIT, whereas CT improved from 63.33 ± 5.16% to 64.84 ± 5.68%. The POST value favored SIT (P = 0.044, d = 0.60; **Figure 3A**).

**Figure 3:**
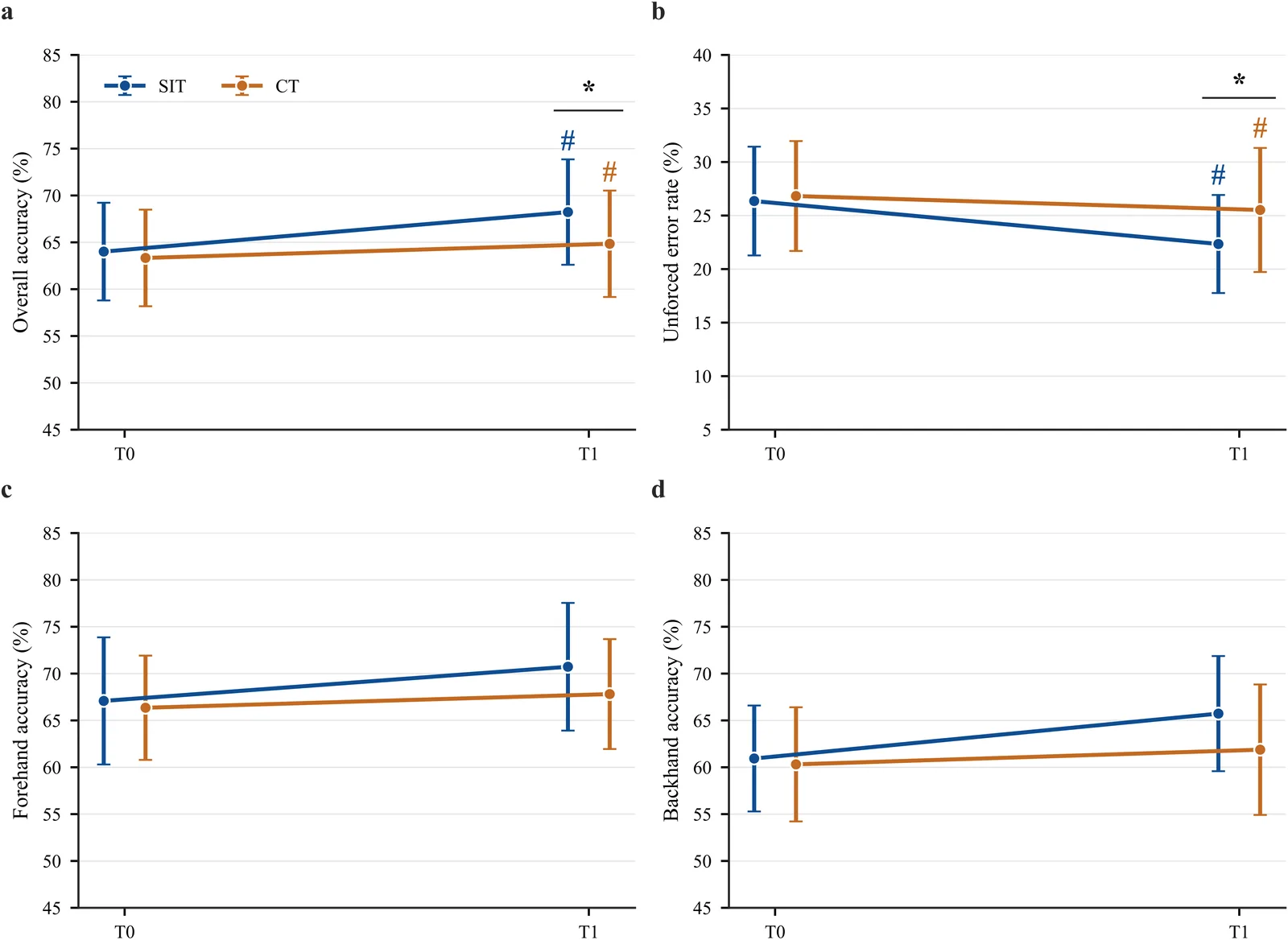
Standardized tennis stroke performance before and after the 8-week intervention. Data are presented as mean ± SD (n = 24 per group). The assessment used an identical programmable ball machine, target zones, ball trajectory, and testing sequence at PRE and POST. Overall stroke accuracy was calculated as successful target hits divided by total valid strokes × 100. Unforced-error rate was calculated as unforced errors divided by total valid strokes × 100. Forehand and backhand accuracy are displayed as descriptive component outcomes. #P < 0.05 versus PRE within the same group; *P < 0.05 for SIT versus CT at POST, where shown. Domain-specific Holm-adjusted group × time interaction results are presented in **Supplementary Table S2**. These interaction tests are distinct from the within-group and POST between-group comparisons indicated by the symbols. Panels show **(a)** overall stroke accuracy, **(b)** unforced-error rate, **(c)** forehand accuracy, and **(d)** backhand accuracy.

The exploratory interaction for unforced-error rate also remained significant after adjustment within the stroke-performance family (F(1,46) = 7.47, unadjusted P = 0.009, adjusted P = 0.009, partial η² = 0.140). The unforced-error rate decreased from 26.35 ± 5.08% to 22.34 ± 4.58% in SIT, compared with a smaller decrease from 26.82 ± 5.13% to 25.52 ± 5.79% in CT. At POST, the SIT group had a lower unforced-error rate than the CT group (P = 0.041, d = −0.61; **Figure 3B**). Forehand and backhand accuracy showed directionally consistent changes and were retained as descriptive component outcomes (**Figures 3C, D**).

### 3.4 Neuromuscular capacity

CMJ height increased over time in both groups (F(1,46) = 39.76, P < 0.001, partial η² = 0.464), from 42.38 ± 1.93 to 44.08 ± 2.45 cm in SIT and from 42.02 ± 1.93 to 43.07 ± 2.43 cm in CT. However, the group × time interaction was not significant (F(1,46) = 2.22, unadjusted P = 0.143, adjusted P = 0.429, partial η² = 0.046), and the POST comparison was also non-significant (P = 0.160, d = 0.41; **Figure 4A**).

**Figure 4:**
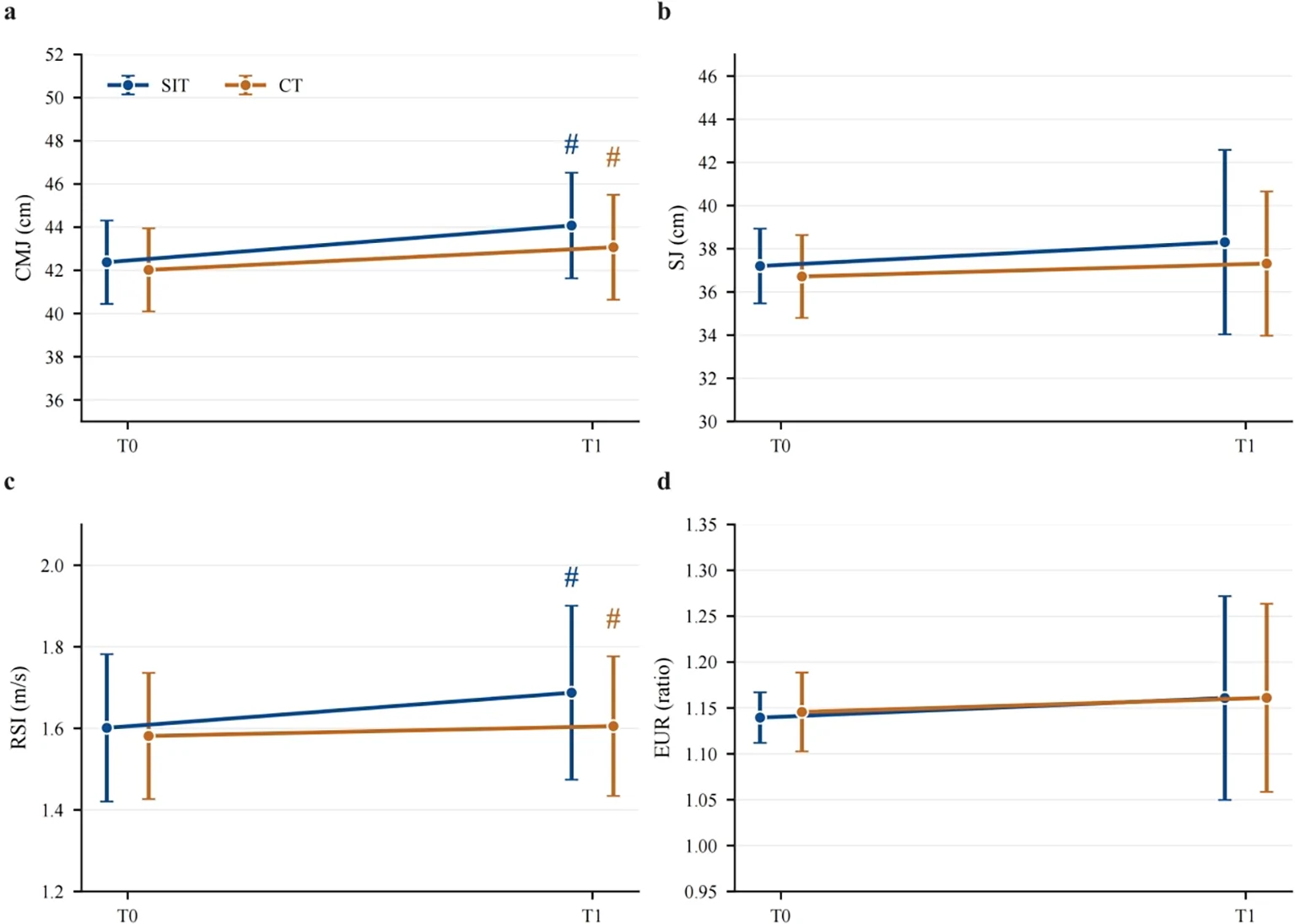
Neuromuscular capacity before and after the 8-week intervention. Data are presented as mean ± SD (n = 24 per group). CMJ and SJ height are expressed in cm; RSI is expressed in m·s^−1^; and EUR is calculated as CMJ height divided by SJ height. RSI was derived from the drop-jump test as jump height divided by ground-contact time. #P < 0.05 versus PRE within the same group; *P < 0.05 for SIT versus CT at POST, where shown. Domain-specific Holm-adjusted group × time interaction results are presented in **Supplementary Table S2**. These interaction tests are distinct from the within-group and POST between-group comparisons indicated by the symbols. Panels show **(a)** countermovement jump height (CMJ), **(b)** squat jump height (SJ), **(c)** reactive strength index (RSI), and **(d)** eccentric utilization ratio (EUR).

The exploratory group × time interaction for RSI remained significant after Holm adjustment within the neuromuscular family (F(1,46) = 17.17, unadjusted P < 0.001, adjusted P < 0.001, partial η² = 0.272). SIT increased from 1.60 ± 0.18 to 1.69 ± 0.21 m·s^−1^, whereas CT changed from 1.58 ± 0.15 to 1.61 ± 0.17 m·s^−1^. Despite this differential longitudinal response, the POST between-group comparison did not reach statistical significance (P = 0.149, d = 0.42; **Figure 4C**). There was limited evidence of differential change in SJ height (F(1,46) = 0.32, unadjusted P = 0.575, adjusted P = 1.000, partial η² = 0.007; **Figure 4B**) or EUR (F(1,46) = 0.04, unadjusted P = 0.836, adjusted P = 1.000, partial η² = 0.001; **Figure 4D**).

### 3.5 Additional physical-performance outcomes

The exploratory analysis supported a greater reduction in 20-m sprint time with SIT after Holm adjustment within the field-performance family (F(1,46) = 7.19, unadjusted P = 0.010, adjusted P = 0.020, partial η² = 0.135). Sprint time decreased from 3.25 ± 0.13 to 3.18 ± 0.16 s in SIT and from 3.27 ± 0.14 to 3.24 ± 0.16 s in CT. However, the POST between-group difference was not significant (P = 0.203, d = −0.37; **Figure 5A**).

**Figure 5:**
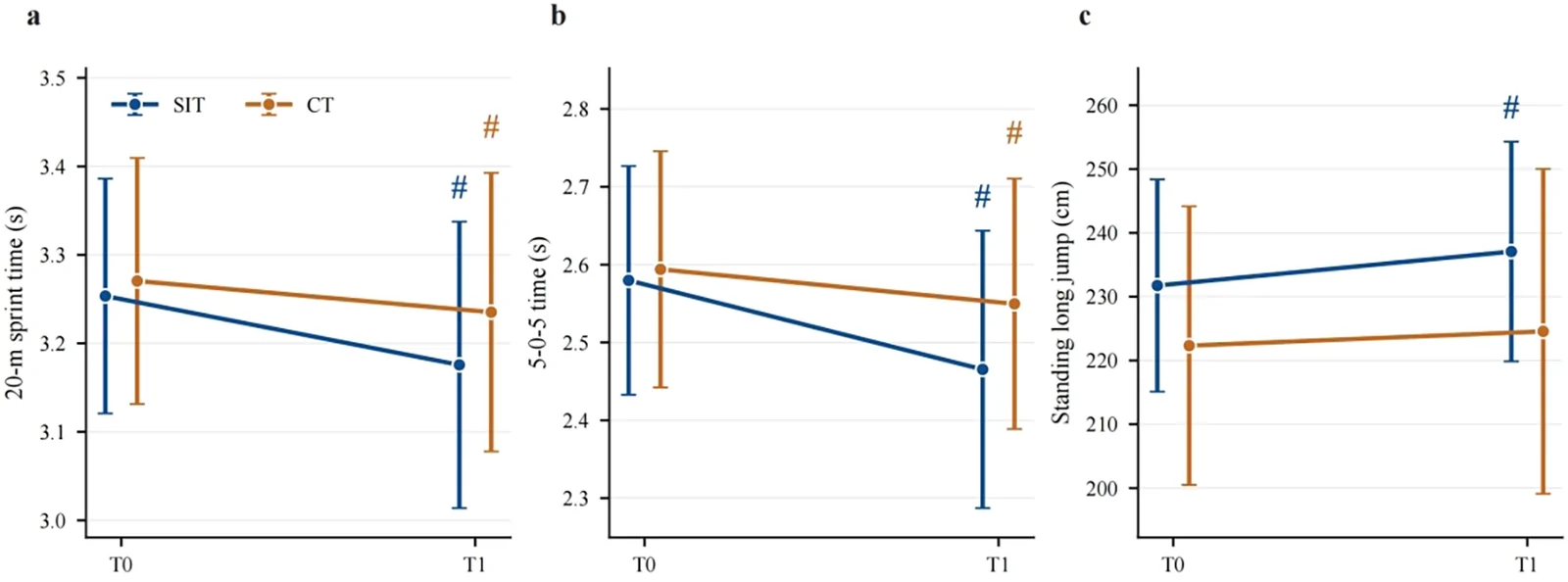
Additional physical-performance outcomes before and after the 8-week intervention. Data are presented as mean ± SD (n = 24 per group). Outcomes include 20-m sprint time, 5-0–5 change-of-direction time, and standing long-jump distance. Lower values indicate better sprint and change-of-direction performance, whereas greater distance indicates better standing long-jump performance. #P < 0.05 versus PRE within the same group; *P < 0.05 for SIT versus CT at POST, where shown. Domain-specific Holm-adjusted group × time interaction results are presented in **Supplementary Table S2**. These interaction tests are distinct from the within-group and POST between-group comparisons indicated by the symbols. Panels show **(a)** 20-m sprint time, **(b)** 5–0–5 change-of-direction time, and **(c)** standing long-jump distance.

The exploratory interaction for 5-0–5 change-of-direction performance also remained significant after adjustment within the field-performance family (F(1,46) = 10.30, unadjusted P = 0.002, adjusted P = 0.007, partial η² = 0.183). SIT improved from 2.58 ± 0.15 to 2.47 ± 0.18 s, whereas CT improved from 2.59 ± 0.15 to 2.55 ± 0.16 s. The POST comparison did not reach significance (P = 0.093, d = −0.50; **Figure 5B**). Standing long-jump performance showed no group × time interaction (F(1,46) = 0.87, P = 0.356, partial η² = 0.019), despite a small overall time effect (F(1,46) = 5.22, P = 0.027, partial η² = 0.102; **Figure 5C**).

### 3.6 Intervention fidelity and training load

Across the 24 prescribed sessions, attendance was 23.00 ± 1.29 sessions (95.83 ± 5.36%) in SIT and 23.04 ± 0.95 sessions (96.01 ± 3.98%) in CT. Full-protocol completion among attended sessions was 96.55 ± 3.42% and 97.62 ± 2.86%, respectively. Valid heart-rate recordings were available for 537 of 552 attended SIT sessions (97.28%) and 539 of 553 attended CT sessions (97.47%). Mean actual main-exercise duration was 16.29 ± 0.33 min·session^−1^ in SIT and 48.52 ± 0.53 min·session^−1^ in CT; corresponding Edwards TRIMP values were 61.39 ± 6.44 and 61.88 ± 5.12 AU·session^−1^. The different intensity distributions are shown in **Supplementary Table S1**. In SIT, mean 110-m repetition time was 30.23 ± 1.45 s, mean speed was 3.65 ± 0.17 m·s^−1^, and sprint decrement was 2.40 ± 0.12%.

## 4 Discussion

The present study examined the effects of an 8-week court-based SIT program compared with CT in collegiate male tennis players. Within the primary cardiorespiratory family, SIT produced greater improvements in VO_2_max and VT2 after Holm adjustment. Domain-adjusted exploratory analyses also supported greater improvements in stroke accuracy, unforced-error rate, RSI, 20-m sprint time, and 5-0–5 change-of-direction time. Recovery-phase blood lactate estimates favored SIT, although the BLa-15 and BLa-R15 interactions did not remain significant after adjustment. Evidence of differential change was limited for VEmax, additional ventilatory indices, resting and peak blood lactate, CMJ, SJ, EUR, and standing long-jump performance. The findings therefore indicate an outcome-specific rather than universal response to SIT (Sloth et al., 2013).

The magnitude and pattern of change are important for interpreting this hypothesis test. Across the 8-week cycle, VO_2_max increased from 50.41 to 53.24 mL·kg^−1^·min^−1^ in SIT (+2.83) and from 49.83 to 51.58 mL·kg^−1^·min^−1^ in CT (+1.75), while VT2 increased by 214.71 mL·min^−1^ in SIT compared with 106.76 mL·min^−1^ in CT. The larger SIT response was accompanied by a lower recovery-phase blood lactate concentration rather than a higher post-exercise peak: BLa-15 decreased from 6.70 to 5.44 mmol·L^−1^, while BLa-R15 increased from 33.97% to 45.78%. Thus, the central finding is that the court-based SIT program produced greater improvements in VO_2_max and VT2, together with exploratory technical and movement-related benefits. The recovery-phase blood lactate estimates suggest a possible additional response, although their statistical support was weakened after within-family adjustment. This interpretation also agrees with a quantitative review showing that SIT can improve aerobic capacity across different sprint formats, while leaving room for protocol-specific differences in the response (Gist et al., 2014).

### 4.1 Cardiorespiratory fitness and ventilatory thresholds

The superior SIT response for VO_2_max and VT2 is consistent with the ability of repeated near-maximal efforts to provide a large central and peripheral stimulus within a short training period. The protocol combined three sets of three maximal 110-m shuttle repetitions, 20 s between repetitions, and 5 min between sets, giving the athletes repeated exposure to rapid acceleration and deceleration interspersed with recovery rather than a single uninterrupted effort. This design is compatible with the broader evidence that interval training can improve VO_2_max at least as effectively as continuous endurance training when intensity is sufficiently high, even when total exercise volume is lower (Milanović et al., 2015). A recent synthesis likewise found that SIT can improve physical performance, although the response is not uniform across speed, power, and endurance outcomes (Hall et al., 2023). The estimated between-group differences in change were 1.073 mL·kg^−1^·min^−1^ for VO_2_max (unadjusted 95% CI, 0.251 to 1.895) and 107.95 mL·min^−1^ for VT2 (43.52 to 172.37). Both interactions remained significant after Holm adjustment within the primary cardiorespiratory family. The POST standardized differences were d = 0.74 and 0.69, respectively, and describe final group separation rather than differential PRE-to-POST change. In an 8-week randomized trial performed three times weekly, high-aerobic interval protocols increased VO_2_max by 5.5% with 15/15-s intervals and by 7.2% with 4 × 4-min intervals compared with lower-intensity training, indicating that repeated exposure to high oxygen flux can be a potent threshold stimulus (Helgerud et al., 2007).

The VT2 result may be particularly meaningful for tennis because the sport requires athletes to repeatedly move between submaximal recovery periods and intense rallies. A rightward shift in VT2 suggests that a greater absolute workload could be sustained before the ventilatory and metabolic costs of exercise rose sharply. This interpretation is compatible with the principle that interval-training adaptations depend on the interaction between intensity, work-interval duration, recovery, and accumulated high-intensity time rather than on the label of SIT alone (MacInnis and Gibala, 2017). The cardiopulmonary programming literature also stresses that work-interval length and recovery determine whether a session emphasizes oxygen delivery, peripheral fatigue, or anaerobic contribution (Buchheit and Laursen, 2013a). Evidence of differential change in VEmax was limited, and neither the VE/VCO_2_ slope nor the VT2/VO_2_max ratio was significant after adjustment within the additional ventilatory family. The findings therefore do not support a generalized improvement in ventilatory efficiency. The most defensible conclusion is a selective enhancement of maximal aerobic capacity and the threshold at which high-intensity work becomes less sustainable, not a uniform remodeling of every cardiopulmonary variable. This interpretation is also consistent with a 6-week running SIT study in which VO_2_max increased by 11.5% (46.8 to 52.2 mL·kg^−1^·min^−1^) while maximal cardiac output did not increase; continuous training, in contrast, increased cardiac output by 9.5%, supporting a possible peripheral contribution to the SIT response (Macpherson et al., 2011).

The size of the response also appears plausible for an eight-week program. A previous controlled study reported similar metabolic and performance adaptations after low-volume SIT and traditional endurance training, despite a marked difference in training time (Gibala et al., 2006). Similar metabolic convergence has also been reported after low-volume SIT and traditional endurance training in humans, supporting the idea that the present advantage may arise from the specific movement and recovery structure rather than from a wholly different adaptation pathway (Burgomaster et al., 2008). These findings provide a plausible physiological context for the present VO_2_max and VT2 responses. The repeated acceleration–recovery demands of shuttle running may also involve contributions from motor-unit recruitment and phosphocreatine resynthesis, although their role in explaining the larger SIT response remains hypothetical.

### 4.2 Blood lactate responses and recovery

Recovery-phase blood lactate estimates favored SIT, although neither interaction remained significant after adjustment within the lactate family. The estimated between-group difference in change was −0.75 mmol·L^−1^ for BLa-15 (unadjusted 95% CI, −1.36 to −0.15; partial η² = 0.119) and 7.59 percentage points for BLa-R15 (1.64 to 13.54; partial η² = 0.125). Both adjusted P values were 0.054. These estimates describe a larger reduction in BLa-15 and a greater relative decline from peak concentration with SIT, while retaining uncertainty after multiplicity. adjustment. Correspondingly, BLa-R15 increased by 11.81 percentage points in SIT and by 4.23 percentage points in CT. This pattern indicates a greater relative reduction in circulating blood lactate concentration between the observed post-exercise peak and the 15-min recovery measurement following SIT. The direction of this finding is consistent with recent comparative work showing more pronounced changes in post-exercise blood lactate responses following interval training than continuous training (Xie et al., 2024). In a 6-week randomized trial involving 16 collegiate tennis players, court-based SIT similarly produced a large within-group improvement in the reported recovery-phase blood lactate measure (ES = 1.03), whereas no significant change was observed in the continuous-training group (Yang et al., 2025).

Recovery-phase blood lactate concentration reflects the integrated influence of exercise characteristics, training status, and recovery conditions. Previous research has shown that training status may affect the post-exercise blood lactate profile and its subsequent change during recovery (Donovan and Brooks, 1983) (Messonnier et al., 2013). In the present study, the lower BLa-15 and higher BLa-R15 following SIT indicate a more pronounced change in circulating blood lactate concentration during passive recovery. The repeated acceleration, deceleration, and reacceleration demands of the shuttle-sprint protocol may have contributed to this response by providing a distinct metabolic stimulus. However, the present findings are most appropriately interpreted at the level of the observed recovery-phase blood lactate profile. Recovery mode should also be considered, as post-exercise blood lactate responses may differ between active and passive recovery conditions (Taoutaou et al., 1996; Menzies et al., 2010; Devlin et al., 2014).

From a tennis-performance perspective, recovery-phase responses may be relevant because repeated high-intensity efforts are interspersed with relatively brief recovery periods. Tennis fatigue reflects the combined accumulation of metabolic, neuromuscular, and perceptual stress, with potential consequences for stroke control and movement quality. Nevertheless, the present blood lactate measurements were obtained during passive recovery following a standardized exercise test rather than during match play. The association between the observed changes in BLa-15 and BLa-R15 and the improvement in stroke performance should therefore be interpreted cautiously. Further research incorporating tennis-specific match conditions is required to determine the practical relevance of these recovery-phase blood lactate responses (Thomas et al., 2005; Brooks, 2009).

### 4.3 Tennis-specific stroke performance

Stroke-performance interactions remained significant after Holm adjustment within the technical-performance family, although these outcomes were exploratory. Overall stroke accuracy increased from 64.01% to 68.23% in SIT (+4.22 percentage points), whereas CT increased from 63.33% to 64.84% (+1.51 percentage points); the interaction was large (partial η² = 0.335). The unforced-error rate also fell from 26.35% to 22.34% in SIT (−4.01 percentage points), compared with a reduction from 26.82% to 25.52% in CT (−1.30 percentage points). These changes indicate improved target accuracy and fewer unsuccessful returns under standardized ball-machine conditions. This is relevant because tennis fatigue has been linked to changes in both physiological readiness and stroke-related performance, rather than to a single isolated fitness variable (Hornery et al., 2007). Earlier tennis research also evaluated stroke quality directly using error rates, ball velocity, and placement precision (Eijnde et al., 2001). Measurement validity is relevant to this interpretation: in 85 young male tennis players, ball-speed reliability was high across strokes (ICC 0.90–0.98), whereas accuracy was comparatively stable across competitive levels (González-González et al., 2018).

Improved conditioning may have contributed to more consistent positioning and stroke execution across repeated ball feeds. Under fixed delivery conditions, successful target placement requires coordination of court movement, racket preparation, and ball contact. Previous findings that fatigue reduces stroke precision and that interval-training design can influence technical performance support a possible contribution of conditioning to these execution demands (Davey et al., 2002; Rodríguez and Soto, 2017). However, the stroke test was performed on a separate visit from the treadmill and lactate assessments and did not impose a late-match fatigue condition. No mediation analysis was performed; therefore, the technical improvement cannot be attributed specifically to altered lactate handling or increased VO_2_max.

Standardized ball delivery and predefined targets provide a consistent basis for comparing stroke execution before and after training. The observed gains therefore concern the accuracy and consistency of prescribed groundstrokes. Competitive tennis additionally requires anticipation of an opponent’s actions, shot selection, and adjustment to changing trajectories and rally situations. Improved execution under predictable feeding conditions addresses one component of these broader demands. Whether the 4.22-percentage-point accuracy increase in SIT persists during opponent-based point play is a relevant question for subsequent research. Previous comparisons of playing and nonplaying aerobic training likewise show that physiological and stroke-performance responses can differ according to the training task (Pialoux et al., 2015).

### 4.4 Neuromuscular and physical-performance adaptations

The neuromuscular results show why the training response should be described as outcome-specific. RSI increased from 1.60 to 1.69 in SIT and from 1.58 to 1.61 in CT. The estimated between-group difference in change was 0.062 m·s^−1^ (unadjusted 95% CI, 0.032 to 0.092; partial η² = 0.272), and the interaction remained significant after adjustment within the neuromuscular family (adjusted P < 0.001). This exploratory result supports a greater improvement in RSI with SIT. The non-significant POST comparison (P = 0.149, d = 0.42) concerns final group separation and addresses a different contrast. RSI is influenced by the ability to rapidly use eccentric loading, coordinate the stretch-shortening cycle, and express force within a short ground-contact period, so a repeated-sprint program may provide a relevant but incomplete stimulus (Ramirez-Campillo et al., 2023). A separate meta-analysis in male athletes also indicates that RSI responds most reliably when the program contains a sufficiently direct reactive-strength stimulus, which was not the primary design of the present intervention (Rebelo et al., 2022). The measurement literature also indicates that RSI can be reliable when the protocol is standardized, with ICC values above 0.90 in a 30-cm depth-jump protocol, although reliability does not guarantee sensitivity to the smallest worthwhile change (Flanagan et al., 2008). Within the proposed framework, RSI is a parallel neuromuscular outcome. Its improvement does not establish that reactive strength was necessary for, or mediated, the improvement in stroke accuracy.

The absence of differential effects for CMJ, SJ, and EUR is therefore not contradictory to the improvement in RSI. CMJ height and SJ height emphasize impulse and concentric force production over a longer movement time, whereas RSI is more sensitive to rapid force expression and short contact time. These measures also have different technical and reliability profiles. A training program dominated by maximal shuttle repetitions can improve the ability to repeat accelerations and tolerate fatigue, but it does not necessarily provide the heavy resistance, eccentric overload, or deliberate plyometric contacts required to maximize jump height or the organization of the stretch-shortening cycle. This is consistent with the broader view that neuromuscular power is highly specific to the force-time demands of the training stimulus (Cormie et al., 2011). The CMJ literature also cautions that jump performance is useful for monitoring neuromuscular status, but that the test should be interpreted in relation to the specific fatigue and adaptation state being monitored (Claudino et al., 2017). In mixed male and female field-sport athletes, two rebound-jump protocols showed ICC values of at least 0.80 and coefficients of variation no greater than 10%, yet their usefulness for detecting the smallest worthwhile change remained questionable (Comyns et al., 2019).

The field tests also showed outcome-specific responses. The estimated between-group differences in change were −0.043 s for 20-m sprint time (unadjusted 95% CI, −0.074 to −0.011) and −0.070 s for 5-0–5 change-of-direction time (−0.114 to −0.026). Both interactions remained significant after adjustment within the field-performance family, with adjusted P values of 0.020 and 0.007, respectively. These exploratory findings support greater improvements in sprint and change-of-direction performance with SIT. Their non-significant POST comparisons concern final group levels and do not test the same contrast. Standing long-jump change was less clearly differentiated between groups, limiting broader claims about lower-limb explosive performance. This interpretation is consistent with the principle that plyometric and sprint adaptations are specific to the loading pattern, contact strategy, and movement velocity trained (Markovic and Mikulic, 2010).

This outcome-dependent pattern has practical value. Court-based SIT may serve as an aerobic conditioning option, with the exploratory analyses also indicating larger PRE-to-POST changes in RSI, sprint time, and change-of-direction time. If the objective is to maximize acceleration, COD, or jump power in isolation, SIT should probably be combined with progressive strength and plyometric training. Such a combined approach would respect the distinction between metabolic conditioning and force-production development rather than expecting one program to optimize every neuromuscular metric. The literature on athletic performance similarly emphasizes that strength and power qualities are related but not interchangeable (Suchomel et al., 2016). Associations between strength, jump performance, and sprint time have also been reported in rugby union players (Furlong et al., 2021). A tennis-specific 8-week combined repeated-sprint and explosive-strength program improved sprint, CMJ, and repeated-sprint ability with effect sizes of 0.56–1.12, while maximal aerobic performance did not change, illustrating the value of pairing sprint work with an explicit power stimulus when the aim is neuromuscular adaptation (Fernandez-Fernandez et al., 2015).

### 4.5 Practical applications and strengths

The intervention offers a feasible option for collegiate tennis programs because it used the court, shuttle movement, and short work-to-recovery sequences rather than requiring specialized laboratory equipment. Three sessions per week over eight weeks produced greater improvements in VO_2_max and VT2 with SIT than with CT. Domain-adjusted exploratory analyses also supported improvements in stroke performance, RSI, sprint performance, and change-of-direction performance, whereas the recovery-phase blood lactate findings remained uncertain after adjustment. Coaches should retain progressive control of total load, because the same high-intensity stimulus that may improve recovery can also amplify neuromuscular fatigue when placed immediately before demanding skill sessions. Programming should therefore account for both cardiopulmonary and neuromuscular load, as the latter can remain high even when the total running volume is low (Buchheit and Laursen, 2013b).

The main strengths were the single-blind randomized two-arm design, the continuous-training comparator, and the joint assessment of physiological, technical, and physical outcomes. Group × time interactions were distinguished from POST group comparisons. The recorded main-exercise duration was shorter in SIT than CT (16.29 ± 0.33 versus 48.52 ± 0.53 min per session; Section 3.6). These values exclude the common warm-up and recovery activities and should not be presented as total session times. They indicate a shorter conditioning component in the tested program, not a measured reduction in athletes’ total weekly commitments or superiority at an equivalent training dose. Previous controlled comparisons likewise support studying training responses in relation to both time commitment and program content (Gillen et al., 2016; Hommel et al., 2019).

### 4.6 Limitations

Several limitations should temper the conclusions. First, the sample comprised 48 collegiate male tennis players classified as highly trained/national-level athletes. Applicability to female athletes, younger players, recreational players, and professional-level athletes remains uncertain because these populations differ from the present sample in demographic characteristics, training background, or competitive demands. Second, the intervention lasted eight weeks; this duration is adequate to detect early physiological adaptation but cannot determine whether the observed advantages persist, plateau, or translate to a full competitive season. Third, the study did not quantify match-play outcomes, rally duration, movement distance, or day-to-day training load outside the prescribed intervention. Consequently, the proposed links among aerobic adaptation, recovery-phase blood lactate, and stroke accuracy remain hypotheses rather than demonstrated associations or mediation effects. The pre–post comparison does not establish the temporal ordering of these adaptations. Mitochondrial adaptations, lactate transport, and phosphocreatine resynthesis were not directly assessed; their proposed contributions therefore represent literature-informed hypotheses rather than mechanisms established by this study. An important limitation is that SIT and CT differed in exercise mode, intensity, and session duration. The observed between-group differences therefore represent the effects of the two programs as prescribed, without separating the contributions of the lower-volume/high-intensity stimulus, exercise mode, and training duration. Sprint number and duration may themselves influence VO_2_max adaptation (Vollaard and Metcalfe, 2017; Vollaard et al., 2017).

Fourth, BLa-R15 was derived from the observed peak and 15-min blood lactate concentrations and therefore summarizes the relative concentration change between two recovery points rather than the complete temporal profile of post-exercise blood lactate. Future studies incorporating kinetic modelling and complementary physiological measurements may provide a more detailed characterization of the recovery-phase response. Fifth, the stroke test, although standardized, did not include the perceptual and tactical demands of competition, and the neuromuscular tests may have been influenced by familiarization and day-to-day readiness. Finally, several outcomes showed significant interactions but non-significant post-training group differences. Future studies should therefore be powered for the smallest practically important between-group difference, include longer follow-up, and combine physiological testing with ecologically valid match and rally analyses (Lambrich and Muehlbauer, 2025). Studies of high-intensity aerobic interval training also show that measurable metabolic changes can occur within a short period, reinforcing the need for longer follow-up before making claims about durable adaptation (Talanian et al., 2007). Participants and supervisors knew the training allocation, so assessor blinding could not eliminate differences in expectations or training behavior. The sample-size calculation did not guarantee sensitivity to small effects across all secondary outcomes, and within-family adjustment does not provide a single error-rate guarantee across the entire exploratory outcome set. The absence of a tennis-training-only group also prevents attribution of absolute PRE-to-POST changes solely to the added conditioning program.

## 5 Conclusion

An 8-week, three-session-per-week court-based SIT program produced greater improvements than CT in VO_2_max and VT2 in collegiate male tennis players classified as highly trained/national-level athletes. Domain-adjusted exploratory analyses also supported greater improvements in accuracy and unforced-error rate during the standardized stroke test, as well as in RSI, 20-m sprint time, and 5-0–5 change-of-direction performance. Recovery-phase blood lactate estimates favored SIT but did not remain significant after adjustment. Several jumping and ventilatory outcomes showed limited evidence of differential adaptation. These findings support the tested court-based SIT program as an aerobic conditioning option for highly trained collegiate male tennis players, with additional exploratory benefits in standardized stroke execution and selected physical-performance tests.

## Data Availability

The raw data supporting the conclusions of this article will be made available by the authors, without undue reservation.
