# Supplementary material for: Effects of 8-week sprint interval training on cardiorespiratory fitness, blood lactate responses, stroke performance, and neuromuscular function in collegiate male tennis players
Source: Front Physiol. 2026 Oct 29;17:1956423. doi: 10.3389/fphys.2026.1956423 (PMC13760609; doi:10.3389/fphys.2026.1956423)
Supplement: Supplementary file 1 [file Table1.docx]

**Supplementary Table S1.**

| **Intervention fidelity, actual sprint performance, and internal training load** | | |
| --- | --- | --- |
|  |  |  |
| *Values are mean ± SD across participants unless otherwise indicated (n = 24 per group).* | | |
|  |  |  |
| **Measure** | **SIT** | **CT** |
| **Intervention fidelity** |  |  |
| Attendance, sessions | 23.00 ± 1.29 | 23.04 ± 0.95 |
| Attendance, % of 24 prescribed sessions | 95.83 ± 5.36 | 96.01 ± 3.98 |
| Full-protocol completion, % of attended sessions | 96.55 ± 3.42 | 97.62 ± 2.86 |
| Valid HR recordings, n/N attended sessions (%) | 537/552 (97.28) | 539/553 (97.47) |
| **Actual main-exercise duration** |  |  |
| Overall, min/session | 16.29 ± 0.33 | 48.52 ± 0.53 |
| Weeks 1–2, min/session | 16.46 ± 0.46 | 29.79 ± 0.58 |
| Weeks 3–4, min/session | 16.16 ± 0.69 | 44.53 ± 1.37 |
| Weeks 5–8, min/session | 16.24 ± 0.36 | 59.74 ± 0.54 |
| **Heart rate and internal load** |  |  |
| Mean HR, beats·min⁻¹ | 162.71 ± 7.37 | 114.65 ± 3.33 |
| Peak HR, beats·min⁻¹ | 192.16 ± 5.40 | 170.62 ± 4.72 |
| Time <50% HRmax, min/session | 0.09 ± 0.09 | 10.10 ± 1.46 |
| Zone 1 (50–60% HRmax), min/session | 0.60 ± 0.42 | 21.49 ± 0.87 |
| Zone 2 (60–70% HRmax), min/session | 2.35 ± 1.05 | 11.73 ± 0.98 |
| Zone 3 (70–80% HRmax), min/session | 2.94 ± 0.56 | 4.53 ± 0.91 |
| Zone 4 (80–90% HRmax), min/session | 4.26 ± 0.36 | 0.83 ± 0.30 |
| Zone 5 (90–100% HRmax), min/session | 6.05 ± 1.87 | 0.00 ± 0.00 |
| Edwards TRIMP, AU/session | 61.39 ± 6.44 | 61.88 ± 5.12 |
| Edwards TRIMP, weeks 1–2, AU/session | 63.17 ± 7.23 | 62.64 ± 5.35 |
| Edwards TRIMP, weeks 3–4, AU/session | 60.68 ± 6.65 | 61.84 ± 5.79 |
| Edwards TRIMP, weeks 5–8, AU/session | 60.79 ± 6.37 | 61.48 ± 5.16 |
| **SIT sprint execution** |  |  |
| Mean 110-m repetition time, s | 30.23 ± 1.45 | Not applicable |
| Mean 110-m speed, m·s⁻¹ | 3.65 ± 0.17 | Not applicable |
| Sprint decrement, % | 2.40 ± 0.12 | Not applicable |

**Table S2**

**Between-group differences in change and domain-specific Holm-adjusted interaction tests**

| Outcome | Difference in change [unadjusted 95% CI] | Interaction partial η² | Unadjusted P value | Within-family Holm P value |
| --- | --- | --- | --- | --- |
| 1 Primary cardiorespiratory outcomes (3 outcomes) | | | | |
| VO₂max (mL·kg⁻¹·min⁻¹) | 1.073 [0.251, 1.895] | 0.130 | 0.011658 | 0.023317 |
| VEmax (L·min⁻¹) | −0.350 [−3.172, 2.472] | 0.001 | 0.803960 | 0.803960 |
| VT2 (mL·min⁻¹) | 107.949 [43.524, 172.373] | 0.198 | 0.001518 | 0.004553 |
| 2 Additional ventilatory indices (2 outcomes) | | | | |
| VT2/VO₂max (pp) | 1.277 [−0.247, 2.801] | 0.058 | 0.098433 | 0.098749 |
| VE/VCO₂ slope | −0.335 [−0.6681, −0.0010] | 0.081 | 0.049374 | 0.098749 |
| 3 Blood lactate responses (4 outcomes) | | | | |
| Resting BLa (mmol·L⁻¹) | 0.011 [−0.037, 0.058] | 0.004 | 0.657612 | 1.000000 |
| Peak BLa (mmol·L⁻¹) | 0.070 [−0.359, 0.499] | 0.002 | 0.744089 | 1.000000 |
| BLa-15 (mmol·L⁻¹) | −0.754 [−1.363, −0.145] | 0.119 | 0.016357 | 0.054063 |
| BLa-R15 (pp) | 7.592 [1.643, 13.542] | 0.125 | 0.013516 | 0.054063 |
| 4 Stroke performance (2 outcomes) | | | | |
| Stroke accuracy (pp) | 2.708 [1.577, 3.840] | 0.335 | 0.000016 | 0.000032 |
| Unforced-error rate (pp) | −2.708 [−4.703, −0.714] | 0.140 | 0.008875 | 0.008875 |
| 5 Neuromuscular performance (4 outcomes) | | | | |
| CMJ (cm) | 0.650 [−0.228, 1.528] | 0.046 | 0.142924 | 0.428773 |
| SJ (cm) | 0.507 [−1.298, 2.311] | 0.007 | 0.574708 | 1.000000 |
| RSI (m·s⁻¹) | 0.0621 [0.0319, 0.0922] | 0.272 | 0.000145 | 0.000580 |
| EUR | 0.0059 [−0.0511, 0.0629] | 0.001 | 0.836167 | 1.000000 |
| 6 Field-based physical performance (3 outcomes) | | | | |
| 20-m sprint time (s) | −0.0426 [−0.0745, −0.0106] | 0.135 | 0.010128 | 0.020255 |
| 5-0-5 COD time (s) | −0.0700 [−0.1139, −0.0261] | 0.183 | 0.002423 | 0.007270 |
| Standing long jump (cm) | 3.086 [−3.576, 9.747] | 0.019 | 0.355959 | 0.355959 |
